# Psychometric validation of the Specific Phobia Questionnaire in an Australian community sample

**DOI:** 10.1080/00049530.2025.2464850

**Published:** 2025-02-13

**Authors:** Jane Mathews, Bethany M. Wootton, Karen Moses

**Affiliations:** a School of Psychology, Western Sydney University, Penrith, New South Wales, Australia; b Discipline of Psychology, Graduate School of Health, University of Technology Sydney, Ultimo, New South Wales, Australia

**Keywords:** Anxiety, fear, interference, specific phobia, Specific Phobia Questionnaire

## Abstract

**Objective:**

The Specific Phobia Questionnaire (SPQ; Fairbrother & Antony, 2012) is a 43-item scale that measures fear and interference towards various specific phobias. This study aimed to investigate the psychometric properties of the SPQ in an Australian population.

**Method:**

The sample consisted of 287 participants, aged 18–76 (*M* = 28.30, SD = 12.07).

**Results:**

Confirmatory factor analysis indicated a good fit of the multidimensional factor structure. The SPQ showed excellent internal consistency in total, fear items, interference items, and low to high internal consistency when classified into the five factors. The SPQ demonstrated adequate convergent and discriminant validity when correlated with the Specific Phobia Dimensional Scale and the Panic Disorder Severity Scale – Self Report, respectively.

**Conclusions:**

Overall, the SPQ is a valid and reliable measure to use in an Australian community population. Future research is considered.

## KEY POINTS

**What is already know about this topic:**

1. Anxiety disorders are among the most experienced mental health conditions in Australia. Of these, specific phobia has the highest lifetime prevalence.
2. Few wide-ranging, reliable and valid assessment tools that align with DSM-specific phobia criteria exist.
3. The Specific Phobia Questionnaire (SPQ) has been psychometrically validated with excellent validity, excellent reliability and fits a multidimensional factor structure based on the DSM-5 with a Canadian population.

**What this topic adds:**

1. Findings from this study demonstrate that the SPQ fits a multidimensional factor structure, has good validity and overall great reliability in an Australian community sample.
2. The SPQ is a useful tool in the assessment of specific phobia in an Australian population.
3. The SPQ can be distinguished from other unrelated anxiety scales.

The Diagnostic and Statistical Manual of Mental Health Disorders – Fifth Edition – Text Revision (DSM-5-TR) defines specific phobia as a marked and persistent fear of an object or situation for at least 6 months that is unreasonable or excessive and leads to significant distress or impairment in functioning (American Psychiatric Association [APA], 2022). The DSM-5-TR includes five distinct categories of specific phobia: (1) animals, (2) natural environment (e.g., heights or water), (3) blood-injection injury (e.g., needles and medical procedures), (4) situational (e.g., elevators or crowds) and (5) other (e.g., vomiting or clowns; American Psychiatric Association, 2022). Specific phobia has a lifetime prevalence at 12.5% in the United States (Kessler et al., 2005) and 7.4% worldwide (Wardenaar et al., 2017). Unfortunately, the prevalence rates of specific phobia are currently unknown in the Australian population. These prevalence rates are a cause for concern as not only are specific phobias often persistent and have an early onset, but they are also strongly predictive of the development of other anxiety disorders (Wardenaar et al., 2017) and major depression (Eaton et al., 2018), which have numerous adverse outcomes for people. These include lower self-efficacy, increased stress, poorer decision-making skills and a poorer quality of life (Essau et al., 2014; Zhang et al., 2018).

Fortunately, treatment for specific phobia has proven effective across varying ages and diverse populations (Eaton et al., 2018). However, it is important to note that the effectiveness of the treatment relies upon an accurate diagnosis (Cook & Décary, 2020; Moses et al., 2020). Misdiagnosis may result in inaccurate intervention, leading to poorer treatment outcomes such as higher attrition rates and less symptomology improvement (Jensen-Doss & Weisz, 2008). Thus, developing and using reliable and valid assessment tools is crucial (Souza et al., 2017). A number of key self-report measures have been used to assess specific phobias, such as the Fear Survey Schedule (Akutagawa, 1956) and the Phobic Stimuli Response Scale (Cutshall & Watson, 2004). Although these questionnaires were particularly useful in assessing fears in both research and clinical settings, some limitations have been noted. Of these limitations, the most significant was that both measures did not contain questions aligning with the current diagnostic criteria, thus making them a less ideal method to assess specific phobias (Hood & Antony, 2012; Ovanessian et al., 2018).

Given the lack of comprehensive, wide-ranging, reliable and valid assessment tools that align with DSM-5-TR specific phobia criteria, the Specific Phobia Questionnaire (SPQ) was designed (Fairbrother & Antony, 2012). It assesses the level of fear of a broad range of phobias and measures the extent to which the fear interferes with daily functioning (Ovanessian et al., 2018). Unlike its predecessors, it can screen for different types of specific phobias and differentiate between individuals with and without specific phobias (Ovanessian et al., 2018). This is a major strength as research has shown that as the number of phobias increases for an individual, clinical severity and the likelihood of comorbidity with other mental disorders increases (Wardenaar et al., 2017).

The SPQ also uses cut-off points to distinguish whether a specific phobia is present, which provides several advantages as it is practical, it can inform treatment and may be more cost-effective as compared to a more dimensional self-report scale (Coghill & Sonuga-Barke, 2012). The cut-off values used to determine individuals that align with DSM-5 specific phobia criteria are eight (of a possible score of 40) for animals, 15 (of a possible score of 36) for natural environment, 20 (of a possible score of 56) for blood-injection injury, four (of a possible score of 32) for situational and six (of a possible score of 8) for “other” phobias (Ovanessian et al., 2018). These cut-off points for the same subscales were found to have a sensitivity ranging from .68 to .82 and a specificity ranging from .63 to .79 (Ovanessian et al., 2018). A strength of the SPQ is that it can be utilized in research and clinical settings and help in the development of interventions (Ovanessian et al., 2018).

In a sample of 310 Canadian women, the SPQ displayed a high internal consistency for fear items and interference items (Fairbrother et al., 2016). Ovanessian et al. (2018) further supported this in a sample of 1031 Canadian adults seeking treatment for anxiety. In this sample, there was high internal consistency for the total questionnaire and the subscales based on the DSM-5 subtypes. Similar evidence of good internal consistency was found in a sample of 67 Canadian adults experiencing symptoms of specific phobia (MacLeod et al., 2022). The SPQ has also demonstrated high test–retest reliability in a sample of 26 Canadian undergraduate psychology students (Ovanessian et al., 2018). Thus, the SPQ has shown excellent reliability in Canadian populations; however, reliability is yet to be assessed in other populations, thus limiting the generalizability of the measure.

The SPQ demonstrated good convergent validity as it was significantly correlated with other specific phobia questionnaires, such as the Fear Survey Schedule and the Phobic Stimuli Response Scale (Ovanessian et al., 2018). Discriminant validity was also established through small correlations between the SPQ and the depression subscale of the Depression Anxiety and Stress Scale-21 (DASS-21) (Ovanessian et al., 2018). These results were later replicated by MacLeod et al. (2022). Despite the SPQ’s excellent validity, it has only been validated in Canadian populations. Thus, this measure should be used with caution for the diagnosis or assessment of specific phobia in other populations (Mayo, 2015).

To date, the factor structure of the SPQ has only been examined in one study. Using the DSM-5 specific phobia types as a framework, Ovanessian et al. (2018) assessed the factor structure of the SPQ and found that the model had good fit: SRMR = .09, CFI = 0.92 and RMSEA = .07, thus establishing a multidimensional factor structure based on the five DSM-5 subtypes in a Canadian adult population. However, similar to the validity and reliability, the multidimensional factor structure of the SPQ is yet to be assessed in other populations.

At present, the SPQ has been shown to fit a multidimensional factor structure based on the DSM-5 and has demonstrated excellent validity and reliability in Canadian populations. However, further research is required to evaluate the SPQ’s generalizability to diverse populations, including with those older in age and from various cultural backgrounds (Ovanessian et al., 2018). To date, no studies have sought to evaluate the psychometric properties of the SPQ in an Australian non-clinical community sample and is required to understand the clinical utility within the Australian context. Finally, given that variation in anxiety symptom expression and prevalence varies across cultures (Barlow, 2002; Wardenaar et al., 2017), further evaluation is required to inform use. Finally, this study will be the first to examine the factor structure in a large community (i.e., non-clinical) sample.

Therefore, the current study aims to investigate the psychometric properties of the SPQ in an adult non-clinical Australian community population. Through this study, we can add to the existing literature about the psychometric properties of the SPQ and assess the questionnaire’s generalizability to other populations in hopes of encouraging future use in Australia. Based on previous studies showing excellent validity and reliability in Canadian populations (Fairbrother et al., 2016; MacLeod et al., 2022; Ovanessian et al., 2018), it is hypothesized that the SPQ will demonstrate (1) a multidimensional factor structure consistent with the five DSM-5 categories of specific phobia. That is, animals, natural environment, blood-injection injury, situational and “other” phobias, (2) good validity and (3) good reliability in an Australian community sample. Method

## Participants

A total of 412 participants commenced the study. Inclusion in the study required participants to be at least 18 years of age and living in Australia. There were no other inclusion or exclusion criteria. Incomplete data or participants that did not meet inclusion criteria were removed from all analyses (*n* = 125). Therefore, 287 participants were included in the final analyses, with ages ranging from 18 to 76 years (*M* = 28.30, *SD* = 12.07). Sample characteristics are outlined in Table 1. The study was approved by the Human Research and Ethics Committee of Western Sydney University (Approval number: H13180).

**Table 1.** Sample characteristics: frequency (*N* = 287) and percentages.

Variable	Frequency	Percentage (%)
Gender		
Female	210	74.2
Marital status		
Single	183	64.0
Married	52	18.2
De Facto	36	12.6
Divorced	11	3.8
Widowed	1	0.3
Separated	3	1.0
Employment status		
Working part-time	82	28.7
Working full-time	78	27.3
Unemployed	12	4.2
Studying	99	34.6
Retired	2	0.7
Full-time Carer	2	0.7
Other	11	3.8
Highest level of education		
School certificate	29	10.1
Trade certificate	17	5.9
Higher school certificate	138	48.1
Bachelor degree	55	19.2
Postgraduate degree	41	14.3
Doctorate	7	2.4
Country of origin		
Australia	223	77.7
New Zealand	2	0.7
Asia	22	7.7
Europe	4	1.4
United Kingdom	6	2.1
North America	3	1
South America	3	1
Middle East	13	4.5
Africa	3	1.0
Other	8	2.8

*Note*. Percentages are based on valid percent.

## Measures

### The Specific Phobia Questionnaire (SPQ)

The SPQ, a 43-item self-reported scale developed by Fairbrother and Antony (2012), was used to measure specific phobias. The scale uses a five-point Likert scale consisting of two dimensions: (1) level of fear and (2) level of interference with one’s daily life. That is, for each stimuli participants are asked to rate “how fearful you are of each situation” and “how much your fear interferes with your life”. Dimensions are measured ranging from 1 (no fear or no interference) to 5 (extreme fear or extreme interference). Consistent with Ovanessian et al. (2018), the following scoring method was used. The fear scores are summed based on the five DSM-5 subtypes: animals (10 items; e.g., dogs), natural environment (9 items; e.g., heavy rain), situational (8 items; e.g., tunnels), blood-injection injury (14 items; e.g., blood tests) and other (2 items; e.g., choking). The total summed score range for each subtype is 0 to 20, 0 to 36, 0 to 32, 0 to 56 and 0 to 8, respectively, with higher scores indicating higher levels of fear. A total score for the SPQ can be achieved by summing scores across each subtype, ranging from 0 to 152, with higher scores indicating higher levels of overall fear. Previous literature has found excellent internal consistency in the total SPQ, the fear items, and the interference items (α = .95, *α* = .93, *α* = .95, respectively; Fairbrother et al., 2016; Ovanessian et al., 2018).

### The Specific Phobia Dimensional Scale (SP-D)

The SP-D is a ten-item measure that assesses the frequency of symptoms and behaviour relating to specific phobias (Lebeau et al., 2012). All items are rated on a five-point Likert scale ranging from 0 (never) to 4 (all of the time), resulting in a total score ranging between 10 and 50. Higher scores indicate a higher frequency of specific phobia symptomology. An example item is “During the past month I have felt anxious, worried, or nervous about these situations” (Lebeau et al., 2012). The SP-D demonstrated excellent internal consistency in the current sample (α = .95), which is consistent with previous literature (α = .95; Lebeau et al., 2012).

### The Panic Disorder Severity Scale – Self Report (PDSS-SR)

The PDSS-SR is a seven-item scale that measures the severity of panic attacks and panic disorder symptoms (Houck et al., 2002). All items are rated on a five-point Likert scale ranging from 0 (none) to 4 (extreme), resulting in a total score ranging between 7 and 35. Higher scores indicate a higher severity of panic disorder. An example item is “How many panic and limited symptoms attacks did you have during the week?” (Houck et al., 2002). The PDSS-SR demonstrated excellent internal consistency in the current sample (α = .92), which is consistent with previous research (*α* = .91; Mahoney et al., 2017).

## Procedure

Data was collected within a larger research study. Participants were recruited via various social media networks, community noticeboards, university student channels and colleagues via email. Participants were invited to share the study with their networks via email. The number of participants from each recruitment source was not monitored. An anonymous link was sent to participants, which directed them to an information sheet and consent form. If consent was received, the participants were directed to complete a demographic questionnaire, the SP-D, the PDSS-SR and the SPQ, in fixed order. Completion of the online questionnaire was estimated to take approximately 20 min.

## Statistical analysis

All analyses were performed using IBM SPSS Statistics Version 26 and R version 4.2.3. Before conducting statistical analyses, assumption testing was completed. The use of nonparametric tests was deemed appropriate as the data exhibited significant positive skewness and multivariate nonnormality. The skewness of the SPQ total score was 1.05 (SE = .14), the skewness of the SP-D was .77 (SE = .14) and the skewness of the PDSS-SR was 1.34 (SE = .14) indicating that the distributions were significantly skewed. The Shapiro–Wilk test also indicated that the distributions were non-normal (SPQ total: *W*  = 0.92, *p*  = <.001; SP-D total: *W*  = 0.91, *p*  = <.001; PDSS-SR total: *W*  = 0.81, *p*  = <.001).

To examine the internal consistency for the measures SPQ, SP-D and PDSS-SR, Cronbach’s α was calculated. The SP-D and PDSS-SR were used to examine convergent and divergent validity with the SPQ, respectively. Due to non-normal data, Spearman’s ρ was used to examine these correlations. These correlations were compared utilizing Fishers *r* to *z* transformation to compare correlations (Lowry, 2023). The strength of correlation coefficients was interpreted in line with Cohen (1992), where 0.10 is “small”, 0.30 is “moderate” and 0.50 is “large”. A power analysis indicated that a minimum sample size of 181 participants was required to assess factor analysis, assuming a medium effect size of .15, alpha of .05 and power of .80.

A confirmatory factor analysis was used to assess the factor structure of the SPQ using diagonally weighted least squares as it is best for non-normal and ordinal data (Li, 2015; Mîndrilă, 2010). The SRMR, RMSEA, CFI and the Tucker – Lewis Index (TLI) were calculated as a marker of goodness-of-fit. An acceptable fit was demonstrated if values were equal to or less than .08 for SRMR and RMSEA and equal to or higher than .90 for CFI and TLI (Brown, 2006).

## Results

### Data cleaning

**Figure 1.**
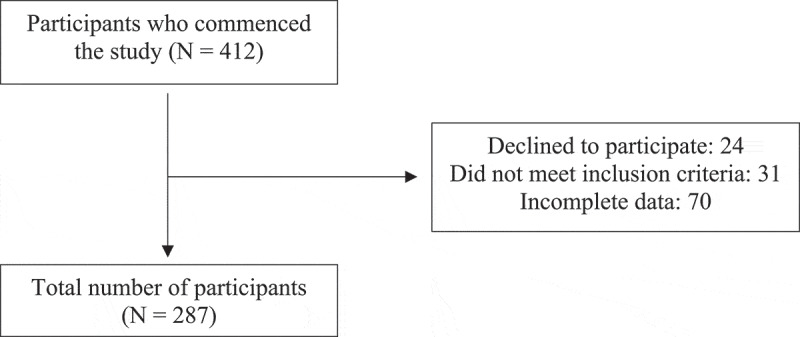
Participant flow diagram.

A total of 412 participants commenced the study, following deletion 287 cases were included in the final study. Twenty-four cases were removed as they did not consent to participate, 31 cases were removed due to not being an Australian resident, and 70 cases were deleted due to the incompletion of the SPQ. Figure 1 illustrates the process. A Missing Values Analysis was conducted, and no missing values were identified.

### Descriptive statistics

Descriptive statistics for each measure are generated in Table 2. Two hundred and thirteen (74.22%) participants scored between 0 and 86 on the SPQ, 66 (23%) scored between 87 and 172, 8 (2.79%) scored between 173 and 258 and 0 (0%) scored between 259 and 344. Based on the cut-off values specified in Ovanessian et al. (2018), the number of participants that met the criteria of each DSM-5 specific phobia subtype are as follows: 128 (44.60%) for animals, 58 (20.21%) for natural environment, 186 (64.81%) for situational, 55 (19.16%) for blood-injection injury and 20 (6.97%) for other.

**Table 2.** Descriptive statistics for measures (*N* = 287).

Measure	*M (SD)*^¶^	Confidence Interval (95%)
SPQ ^†^ – all items	61.04 (45.68)	55.74–66.35
SPQ ^†^ – fear items	36.46 (26.54)	33.37–39.54
SPQ ^†^ – interference items	24.59 (22.44)	21.98–27.20
SPQ ^†^ – animals	8.46 (7.27)	7.62–9.30
SPQ ^†^ – natural environment	8.61 (6.86)	7.81–9.40
SPQ ^†^ – situational	6.25 (5.74)	5.58–6.92
SPQ ^†^ – blood-injection injury	11.12 (11.09)	9.83–12.41
SPQ ^†^ – other	2.02 (1.94)	1.80–2.25
SP-D^‡^	22.33 (10.83)	21.08–23.59
PDSS-SR^§^	3.74 (4.62)	3.21–4.28

*Note*. ^†^ The Specific Phobia Questionnaire. ^‡^ The Specific Phobia Dimensional Scale. ^§^ The Panic Disorder Severity Scale – Self Report. ^¶^ Mean (Standard Deviation).

### Reliability and validity

The SPQ in total, the fear items, and the interference items all demonstrated excellent internal consistency (α = .97, α = .95, α = .94, respectively). The Cronbach’s alpha for the DSM-5 subtypes was as follows: animals (α = .86), natural environment (α = .83), situational (α = .83), blood-injection injury (α =.92), and other (α = .59). Although the subtype “other” was less than the required .70 cut-off in the present study, it was retained for the sake of consistency with Ovanessian et al. (2018) study. Good convergent validity was established through correlations between the SPQ and SP-D (*r* = .54, *p* < .001). The SPQ and PDSS-SR were found to have a weaker positive correlation (*r* = .45, *p* < .001); however, there was no significant difference between the two correlations (*p* = .07), thus discriminant validity could not be established. These latter correlations were compared utilizing Fishers *r* to *z* transformation to compare correlations (Lowry, 2023).

### Assumptions of confirmatory factor analysis

Assumptions of multivariate normality, adequate sample size, the correct a priori model specification, and random sampling were checked. Visual inspection of histograms indicated that normality was not met as each variable showed a positive skew. The second and fourth assumption was met as the final sample was obtained through random sampling and consisted of more than 200 participants. The DSM-5 specific phobia subtypes were used as a framework for the model, which met the third assumption. Six outliers were identified as per being three standard deviations away from the mean: three in the PDSS-SR and three in the total SPQ. The use of Spearman’s rank-order coefficients meant that all outliers could be retained as it is robust against outliers (Schober et al., 2018). Prior to conducting the confirmatory factor analysis, three outliers were identified in the SPQ fear items. However, these were retained as diagonally weighted least squares as the estimator for the confirmatory factor analysis is less sensitive to outliers and non-normality (Li, 2015; Mîndrilă, 2010).

### Confirmatory factor analysis

The confirmatory factor analysis results indicated an excellent model fit for the SPQ fear items (χ^2^(850) = 1244.93, *p* < .001, RMSEA = .05, CFI = .96, TLI = .96, SRMR = .08). Although the chi-square and degrees of freedom are reported, this was not used to assess the goodness-of-fit as it is sensitive to sample size. Table 3 shows the factor loadings and squared multiple correlations for each item in the confirmatory factor analysis model. As shown, items appeared to load well onto the expected factors (with the exception of item 1), and this was statistically significant (*p*  < .001). Although item 1 had a lower factor loading, it was not removed due to the model meeting the goodness-of-fit indices with item 1 retained and to allow better comparability with previous studies. The strength of the correlations ranged from small to moderate (*r* = .16–.74).

**Table 3.** Factor loadings and squared multiple correlations for SPQ ^†^ fear items (*N* = 287).

SPQ ^†^ Items	Unstandardized	Standardized	*p*-value	Squared multiple correlations
Factor 1 (Animals)				
Item 3	1.00	0.52	<.001	0.27
Item 6	0.74	0.55	<.001	0.30
Item 8	0.97	0.58	<.001	0.33
Item 11	1.39	0.63	<.001	0.40
Item 30	0.98	0.61	<.001	0.37
Item 31	1.57	0.69	<.001	0.48
Item 36	1.50	0.61	<.001	0.37
Item 37	1.59	0.72	<.001	0.52
Item 39	1.08	0.61	<.001	0.37
Item 43	1.65	0.63	<.001	0.39
Factor 2 (Natural Environment)				
Item 1	1.00	0.45	<.001	0.21
Item 2	1.23	0.53	<.001	0.28
Item 13	1.34	0.66	<.001	0.43
Item 14	1.03	0.58	<.001	0.33
Item 23	1.64	0.68	<.001	0.47
Item 27	1.43	0.60	<.001	0.36
Item 32	1.69	0.67	<.001	0.44
Item 33	1.23	0.63	<.001	0.39
Item 38	0.84	0.53	<.001	0.28
Factor 3 (Situational)				
Item 4	1.00	0.71	<.001	0.50
Item 7	0.86	0.60	<.001	0.36
Item 16	1.11	0.61	<.001	0.37
Item 22	0.98	0.53	<.001	0.28
Item 26	0.92	0.58	<.001	0.34
Item 29	1.04	0.71	<.001	0.50
Item 34	0.89	0.57	<.001	0.32
Item 40	0.87	0.60	<.001	0.36
Factor 4 (Blood-Injection Injury)				
Item 5	1.00	0.68	<.001	0.47
Item 9	1.05	0.71	<.001	0.51
Item 10	1.23	0.76	<.001	0.58
Item 15	0.75	0.61	<.001	0.37
Item 17	1.09	0.66	<.001	0.44
Item 18	0.71	0.66	<.001	0.44
Item 19	0.91	0.61	<.001	0.38
Item 21	0.95	0.68	<.001	0.46
Item 24	0.98	0.64	<.001	0.41
Item 25	0.95	0.65	<.001	0.43
Item 28	0.78	0.65	<.001	0.42
Item 35	1.25	0.77	<.001	0.59
Item 41	0.96	0.58	<.001	0.34
Item 42	1.16	0.69	<.001	0.48
Factor 5 (Other)				
Item 12	1.00	0.69	<.001	0.48
Item 20	0.76	0.62	<.001	0.38

*Note*. ^†^ The Specific Phobia Questionnaire.

## Discussion

The SPQ is a measure that assesses the level of fear and interference with daily functioning of 43 different fears (Fairbrother & Antony, 2012). The present study aimed to assess the psychometric properties of the SPQ in an adult Australian non-clinical community sample. It was hypothesized that the SPQ would demonstrate (1) a multidimensional factor structure that is consistent with the DSM-5 categories of specific phobia, (2) good validity and (3) good reliability within an Australian community sample.

Hypothesis one was supported as the present study found that the SPQ met the goodness of fit indices, thus displaying a multidimensional factor structure aligning to the DSM-5 specific phobia subtypes: animals, natural environment, situational, blood-injection injury and other. This result is consistent with Ovanessian et al. (2018) confirmatory factor analysis findings. Upon inspecting the factor loadings of the 43 items, only one item, item 1, had a standardized factor loading below .50, which is the suggested cut-off for removal from the scale (Hair et al., 2009). Item 1 refers to “high open places”. Item 1 was also found to have a low factor loading in Ovanessian et al. (2018) study. Overall, however, the findings from the present study are inconsistent with their study as they found several items loaded below .50 to their respective factor: four items in animals, eight items in natural environment, four in situational, five in blood-injection injury and one in other (Ovanessian et al., 2018). Importantly though, comparison between these two studies is difficult given that this study utilized a non-clinical community sample, whilst the former used a clinical sample. Future studies should seek to examine this further by investigating measurement invariance across countries. For example, words or ideas may vary across cultures and languages, particularly between western and non-western cultures, which may explain variances seen across countries (Marques et al., 2011).

Alternatively, the differences in factor loadings in the current study and previous studies (i.e., Ovanessian et al., 2018) may be in part accounted for by recruitment taking place during the COVID-19 pandemic. During this time, there was a significant increase in mental health problems. For example, the global prevalence of anxiety increased by 25% within the first year of the pandemic (World Health Organization, 2022), which may result in increased scores across all items. Additionally, participants may also have been more likely to respond differently during this time on items which focus on health-related outcomes (e.g., “visiting a hospital” or “developing an illness”) or being in an enclosed space with other people (e.g., “elevators” or “flying in an airplane”), which may have a higher perceived risk of catching the COVID-19 virus.

Finally, the Canadian sample differed to the present study in relation to their recruitment source. Participants within the Canadian sample were seeking treatment for anxiety at an outpatient facility, whereas this study comprised a non-clinical Australian community sample. It is not known what kind of anxiety disorders the participants in the Canadian sample were seeking treatment for, but it may be that the participants had anxieties that were not related to specific phobia, hence producing lower factor loadings. Given that there are currently only two confirmatory factor analyses on the SPQ, and these studies have very different recruitment sources, it is difficult to draw any conclusions about the reasons for these differences and further research is required.

Hypothesis two was supported as the SPQ displayed good convergent and discriminant validity with the SP-D and PDSS-SR, respectively. Previous studies found similar findings as the SPQ demonstrated good convergent validity with other scales that also measured specific phobias (MacLeod et al., 2022; Ovanessian et al., 2018). This study was the first to assess the SPQ’s discriminant validity with an unrelated anxiety scale. Its ability to have adequate discriminant validity when correlating with the PDSS-SR illustrates that the SPQ can be distinguished from other unrelated anxiety scales despite comprising common themes associated with anxiety.

Hypothesis three was partially supported. The correlations between items in each of the five factors of the SPQ all falling within the expected range delineated by Paulsen and BrckaLorenz’s (2017) study (.15 to .85 for adequate reliability of a construct) serves in support of hypothesis three. Prior research has not reported on inter-item correlations; thus, comparisons between other research cannot be made. However, correlations between factors in Ovanessian et al. (2018) study were within the expected range. Future studies psychometrically validating the SPQ should include inter-item correlations to assess the reliability of the construct in different populations.

Moreover, when testing Hypothesis 3, excellent internal consistency was found in the total SPQ, the fear items only, and the interference items only. This finding is consistent with previous literature (Fairbrother et al., 2016; Ovanessian et al., 2018). However, the Cronbach’s alpha for the DSM-5 subtypes ranged from good to excellent internal consistency, except for “other”, which was below the .70 cut-off. Hence, hypothesis three was partially supported. It is important to note that this low value may be due to the small number of items in the “other” factor (*N*  = 2) as Cronbach’s alpha has a positive relationship with the number of items (Hoekstra et al., 2018). However, MacLeod et al. (2022) found good to excellent internal consistency for all subtypes despite the small number of items. This finding may have developed due to the sample consisting of adults experiencing symptoms of specific phobia as opposed to the present study, which consisted of a community sample. As above, researchers aiming to validate the SPQ should consider different populations with varying cultures and contexts to gather a wide range of data and increase the generalizability of the questionnaire.

It was observed that mean scores on the SPQ were elevated when compared to Ovanessian et al. (2018) study (fear items only, *M*  = 27.28 and *SD*  = 20.96; interference items only, *M*  = 16.46 and *SD*  = 21.86) and lower when compared to MacLeod et al. (2022) findings (total SPQ, *M*  = 101.22 and *SD*  = 64.29; fear items only, *M*  = 55.76 and *SD*  = 32.29; interference items only, *M*  = 44.46 and *SD*  = 33.18). The lower mean scores found when compared to MacLeod et al. (2022) are unsurprising given that their sample consisted of adults experiencing symptoms of specific phobia. However, it is unexpected that the present study’s mean scores were elevated in comparison to Ovanessian et al. (2018), as it would indicate that the Australian community sample reported a greater level of fear and interference with specific phobias than the Canadian sample seeking treatment for anxiety. Due to the lack of pre-existing studies examining the prevalence of specific phobias within an Australian context, it is difficult to comment on whether this finding is reflective of the general Australian population. Further, our study’s elevated mean score may be in part due to the recruitment process coinciding with the start of the COVID-19 global pandemic, where individuals experiencing an elevated state of anxiety may have been more inclined to participate in our research. In addition, the elevated mean score may also be due to the higher female-to-male ratio in the Australian sample compared to the Canadian sample as Wardenaar et al. (2017) found a higher prevalence of specific phobias in females than males.

There are several strengths associated with this study. First, the present study is only the second study to report on the psychometric properties of the SPQ. Given that there are few wide-ranging, reliable and valid assessment tools that align with DSM diagnostic criteria for specific phobia currently available, this study is an important step in supporting its evidence-based use in everyday clinical practice. Second, this study utilized a diverse community sample, where country of birth (Australian Bureau of Statistics, 2021a) and education and employment (Australian Bureau of Statistics, 2021b) were found to be consistent with that of the general population. This suggests that the SPQ is suitable for use in an Australian population. In addition, this study was the first to evaluate the SPQ’s factor structure outside of the developers of the questionnaire, and the first to examine the factor structure of the SPQ in a large non-clinical community sample.

Notwithstanding these important findings, a number of limitations should be noted. Whilst a diverse sample similar to that of the Australian population was achieved, given that most participants were female and under 25 years of age, the generalizability of findings to the Australian population broadly is limited. Importantly though, this high proportion of female participants is consistent with studies of a similar nature utilizing clinical and non-clinical samples (DeSousa et al., 2017; Moller & Bogels, 2016) and may be reflective of the gender variation of this disorder within the Australian population, which may have also led to a selection bias. Future research may wish to consider strategies to attract a more diverse sample. Secondly, this study did not assess the SPQ’s sensitivity and specificity; thus, future research should consider using clinical and non-clinical Australian samples to examine the sensitivity, specificity and cut-off scores. Another limitation is that the exact number of participants from each recruitment source is unknown as it was not monitored. Future research should consider monitoring recruitment source. Moreover, there appeared to be a high number of participants in the sample that met the cut-off values for the animals and situational subscale. Once again, this finding may be linked to recruitment during the COVID-19 pandemic. The prevalence rate appears to be considerably higher than the 17% of Australians that experienced an anxiety disorder between December 2019 and July 2020 (which was the first year of the COVID-19 pandemic; Australian Bureau of Statistics, 2022). However, it is important to note that this study did not involve conducting a diagnostic interview, therefore a definitive diagnosis cannot be confirmed. It should be noted that the exact prevalence of specific phobia in Australia is currently unknown; thus, future research regarding this is essential.

Overall, the present study adds to the growing literature exploring the psychometric properties and factor structure of the SPQ. The findings demonstrate that the SPQ fits a multidimensional factor structure, has good validity and overall great reliability, except for one subscale, in an Australian community sample. These findings provide valuable insight into the use and applicability of the SPQ within an Australian population. However, it is noted that more research is required utilizing Australian samples to address the limited generalizability at present.

## Data Availability

The data that supports the findings of this study are available from the corresponding author, KM, upon reasonable request.
